# African tick bite fever in returning Swedish travellers. Report of two cases and aspects of diagnostics

**DOI:** 10.1080/20008686.2017.1343081

**Published:** 2017-08-02

**Authors:** Kenneth Nilsson, Katarina Wallménius, Pernilla Rundlöf-Nygren, Susanne Strömdahl, Carl Påhlson

**Affiliations:** ^a^ Department of Medical Sciences, Section of Clinical Microbiology, Uppsala University, Uppsala, Sweden; ^b^ Department of Medical Sciences, Section of Infectious Diseases, Uppsala University, Uppsala, Sweden; ^c^ Centre of Clinical Research, Falu Hospital, Falun, Sweden

**Keywords:** ATBF, rickettsioses, PCR, serology, isolation

## Abstract

**Introduction**: African tick-bite fever, caused by *Rickettsia africae*, is endemic in rural areas of sub-Saharan Africa and a possible cause of fever in returning Swedish travellers. Two patients are presented, and the advantages and disadvantages of different diagnostic methods are discussed.

**Patients and methods**: Two middle-aged men fell ill with fever after returning home from South Africa. Both had single eschars and one also presented with a lymph node swelling. Samples were taken for serology, general bacterial culture from the wound (Patient 1) using a swab and additionally for Patient 2 PCR of a skin biopsy from the eschar.

**Results and discussion**: Both patients seroconverted one month after onset. Real-time PCR of the biopsy was positive, where sequencing of the *gltA* gene was 99–100% consistent with *R. africae*. A drop of fluid from the biopsy contained a sufficient number of bacteria to also allow for isolation of rickettsiae in Vero cell culture. Direct molecular detection by PCR from a swab used for bacteria culture from the eschar from Patient 1 also yielded a positive result. In conclusion, the findings highlight the usefulness of swabs for early non-invasive diagnosis of African tick-bite fever in febrile travellers.

## Introduction

The first isolation of *Rickettsia africae* was in 1992, in a patient who had been bitten by a tick and who presented fever, eschar and lymphadenopathy.[1,2] African tick-bite fever (ATBF) was initially considered to be caused by *R. conorii*, but it is now accepted that two distinctive tick-transmitted diseases, ATBF and Mediterranean spotted fever, coexist within southern Africa.[3,4] *R. africae* is mainly transmitted by non-host-specific hard ticks (*Amblyomma hebraeum* and *Amblyomma variegatum*), which often infest wild ungulates and cattle, but also feed readily on humans, often with multiple inoculation eschars.[1,5] *R. conorii*, on the other hand, is transmitted by the brown dog tick (*Rhipicephalus sanguineus*) and is mostly found in urban areas.[1,5]

At present, ATBF is considered the predominant rickettsial disease in Southern Africa, with seroprevalence against *Rickettsia* spp. up to 50% in healthy rural populations.[6] Jensenius et al. previously reported an incidence of ATBF of 4–5.3% among Norwegian returning travellers, and in other investigations of international travellers it has been found to be the second most frequently identified aetiology, after malaria, among travellers returning from sub-Saharan Africa.[7,8] ATFB is usually a benign disease, but a more severe course has been described among elderly populations.[9]

South Africa is a popular destination among Swedish travellers, and it is common for them to seek outdoor experiences, such as visits to some of the country’s nature reserves. In an earlier review of cases of spotted fever rickettsioses diagnosed serologically with *R. conori* antigen at the Public Health Agency of Sweden, about half of the cases were associated with travel to South Africa.[10]

In the present article, we report on two cases of *R. africae* infection among Swedish travellers returning from South Africa and discuss the symptomatology as well as diagnostic opportunities and challenges. We also provide a review of the literature.

## Case reports and methods

### Patients

*Patient* 1 is a 56-year-old man who had previously undergone surgery for a pituitary tumour but was otherwise healthy. He had stayed two weeks in Cape Town and visited Kruger Park for four days during that trip. He felt ill the day he came home to Sweden, presenting with a fever of 37.8°C, general feelings of being ill, sweaty and hot. Examination at the Clinic of Infectious Diseases, Uppsala University Hospital, in August 2015, six days after disease onset, showed normal status. In addition, a small crust-covered wound with surrounding redness on the left half of the thorax was noticed. Laboratory tests revealed slightly elevated C-reactive protein (59 mg l^–1^), slightly decreased white blood cell count (2.9 × 10^9^ l^–1^), and platelet cell count values (131 × 10^9^  l^–1^), and normal blood smear. Immunochromatographic rapid test for malaria (BinaxNOW® Malaria) for detection of *Plasmoidum* antigens was negative. Liver enzymes were normal. Blood samples were taken for serology against rickettsioses, dengue fever, as well as general bacterial culture from the wound using a swab.

*Patient 2* is a 41-year-old previously healthy man, apart from having mild asthma symptoms. He had travelled in Zimbabwe for 11 days in July 2016, visiting both rural and urban environments. He got several mosquito bites during the trip. After returning home, he stayed for some days in central Sweden, where he was also mosquito-bitten; he even got a tick bite on his right leg. Five days after returning home, he fell ill with malaise and a 38°C fever. He also noticed painful swelling in his right groin. Examination at the Clinic of Infectious Diseases, Uppsala University Hospital, showed a tender and slightly enlarged, about 1.5 cm in diameter, lymph node in the right groin. On the right lower leg, a bite mark was seen, a wound about 2 mm in diameter with redness and raised edges. C-reactive protein was 14 mg l^–1^, white blood cell count 5.4 × 10^9^ l^–1^ and platelet cell count slightly decreased (104 × 10^9^ l^–1^). Malaria microscopy and rapid test were negative. On suspicion of rickettsia infection, a punch biopsy was taken from the wound on his leg and sent for PCR analysis. Serology for rickettsioses and dengue fever was performed four days after onset, and after four weeks a further serum was taken for analysis of rickettsia antibodies.

### DNA extraction and PCR

In Patient 1, DNA was extracted from exudate from the wound swab mixed in saline, and in Patient 2 from the tissue biopsy and later from the isolate obtained from Vero cell culture, using the NucliSENS easyMAG automated extraction platform (bioMérieux, Durham, NC, USA), according to the manufacturer’s instructions. The spotted fever group of *Rickettsia* was assayed using a genus-speciﬁc real-time PCR with probe and primers targeting the citrate synthase-encoding gene (*gltA*), as previously described.[11] The real-time PCR assay was performed in a Rotor-Gene 3000 (Corbett Research, Sydney, Australia) using LC Taqman Master kit (Roche, France). As a negative control, sterile water was included in each ampliﬁcation trial. A plasmid constructed by cloning the sequence from the target gene into a pCR4-TOPO-TA vector (Invitrogen) was used as a positive control.

The SFR-positive samples were further analysed using conventional and semi-nested PCR assays that amplify the 17-kDa, *ompB* and *gltA* genes, as previously described.[12–14] PCR reactions were performed in a GeneAmp® PCR System 9700 (Applied Biosystems, Foster City, CA, USA), and expected PCR products were confirmed using gel electrophoresis (1% agarose) stained with GelRed™ (Biotium Inc., Fremont, CA, USA). PCR products were sent for Sanger sequencing at Macrogen Inc. (Macrogen Europe, Amsterdam, Netherlands). Sequence alignments and analysis were performed using DNA Baser version 2.80.0 (Heracle Software Lilienthal, Germany) and BioEdit Sequence Alignment Editor Version 7.0.5.3 (Ibis Therapeutics, Carlsbad, CA, USA). For species identification, similarities and differences between sequences were examined using the Basic Local Alignment Search Tool (BLAST).

### Serology

As the bacterial antigen for an immunofluorescence assay (IFA), Vero cells infected by *R. helvetica* and supplemented with 10% yolk sac solution were used, as previously described.[15,16] IgG titres ≥128 and/or IgM titres ≥ 64, and/or a fourfold increase in two sera within a 2–4 week interval are considered indicative of infection by *Rickettsia* spp., if homologous antigens are used in the IFA test. When a heterologous antigen is used, a step lower titre may also be indicative of infection.[17] As a negative control, previously tested serum from a healthy blood donor was used and as a positive control, a serum from a patient with proven IgG and IgM end-point titres of 1/128 and 1/256, respectively, to *R. helvetica*. When *R. africae*, in Patient 2, had been cultivated in Vero cells, the culture was used to prepare IFA glass, and the serologic response to *R. helvetica* was, thereafter, compared to *R. africae* as antigen.

### Western blot

The convalescent sera from Patient 2 was diluted to titre 1/200 and tested for WB with *R. helvetica* and *R. africae* whole-cell antigen using Amersham WB system (GE Healthcare Sverige AB, Danderyd, Sverige), in accordance with the manufacturer’s instructions. Serum from a patient previously proven to have a rickettsial infection with high antibody titres in IFA (IgG 1/128) was used as the positive control. A serum from a healthy blood donor and the secondary antibody alone served as negative controls.

## Results

**Figure 1.**
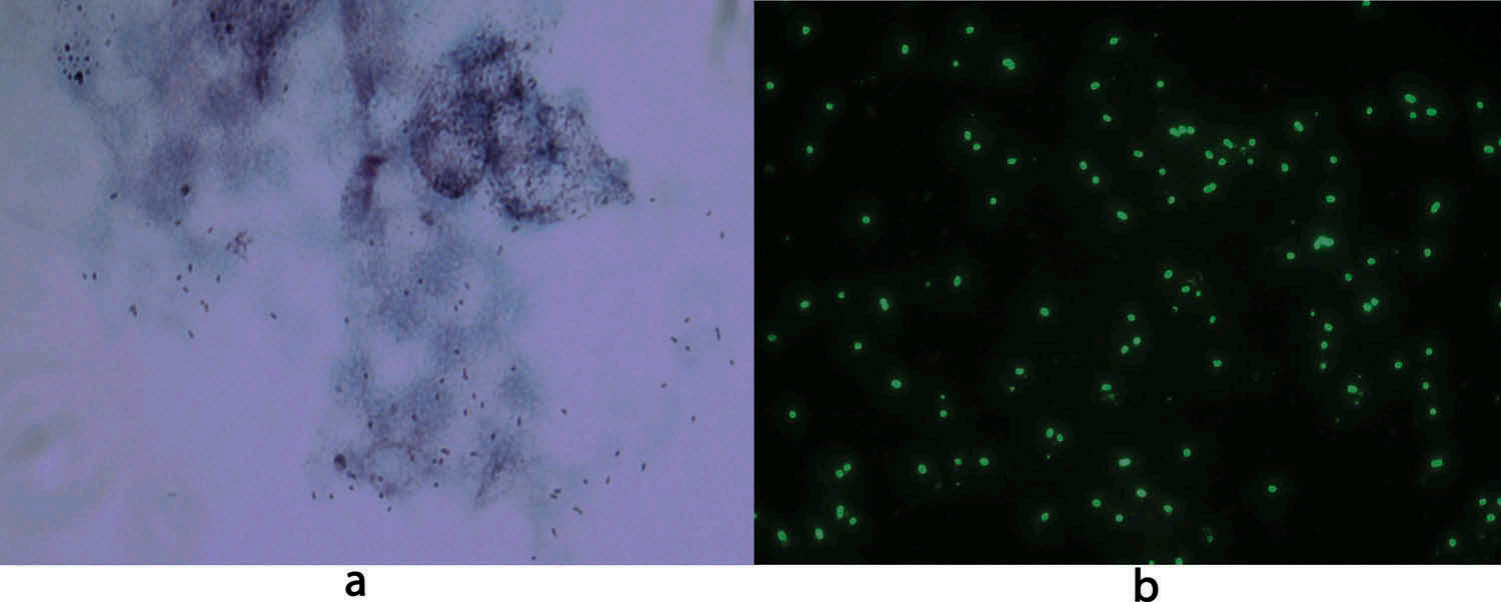
*R. africae* infected Vero-cells showing rickettsial organisms stained red with Gimenez (a) and green with immunofluorescent anti-rickettsial goat anti-rabbit immunoglobulin (b). Original magnification × 1000.

For both patients, real-time PCR was positive for spotted fever rickettsia DNA with a TaqMan-probe-produced amplicon. Patient 1 had a negative bacterial culture from the wound. The remaining amount of wound fluid persisting in the swab gave a very strong PCR reaction, with Ct values of about 24, corresponding to around 5000 copies/PCR reaction. For Patient 2, the PCR of the biopsy also gave a strong reaction, with a Ct value of about 25, which is equivalent to approximately 2000 copies/PCR reaction. For each DNA extraction, 60 µl was eluted, and 5 µl used as the template for the PCR, hence the total amount of bacteria extracted from the biopsy and swab was approximately 12 times higher than the copy number in the PCR. Nested PCR of the *glt*A genes yielded fragments that matched 99–100% with the corresponding gene sequence of *R. africae* (GenBank accession number KJ645939.1). From the biopsy sample from Patient 2 after the DNA extraction, a drop of bloody fluid was left. The drop was mixed with DMEM essential medium and inoculated on Vero cell culture, after which time the 25 cm^2^ vial was centrifuged at 200 × g. After seven days of culture, cytopathic effects could be discerned in the cells, and *Rickettsia* bacteria could be visualized both within Gimenez stain and with immunofluorescence with anti-rickettsia polyclonal rabbit antiserum as the primary antibody (Figure 1). After about one week of culture, a sample of cells and fluid was analysed using real-time PCR; the result was highly positive.

Serology (IFA) with *R. helvetica* as antigen showed IgG and IgM titres of <1/64 and 1/128, respectively, in the early phase serum for Patient 1. The corresponding findings for Patient 2 were <1/64 (G/M) in the early phase serum and 1/128 and 1/256 eight weeks later. When comparing the titres for both patients by use of *R. africae* as antigen, the titres were the same or one titration step higher (Table 1). All negative controls were negative. Analysis of dengue fever was negative for both patients.

**Table 1.** Summary of PCR results and immunofluorescent titres of acute and convalescent sera from Patients 1 and 2 using *R. helvetica* and *R. africae* as antigen, respectively.

Antigen	IFA titres/PCR Patient 1			IFA titres /PCR Patient 2					
	Serum 1 G M		PCR swab	Serum 1 G M		Weeks s1-s2	Serum 2 G M		PCR biopsy
***R. helvetica***	<1/64	1/128	-	<1/64	<1/64	4	1/64	1/256	-
***R. africae***	1/64	1/128	Pos	1/64	1/64		1/128	1/256	Pos

**Figure 2.**
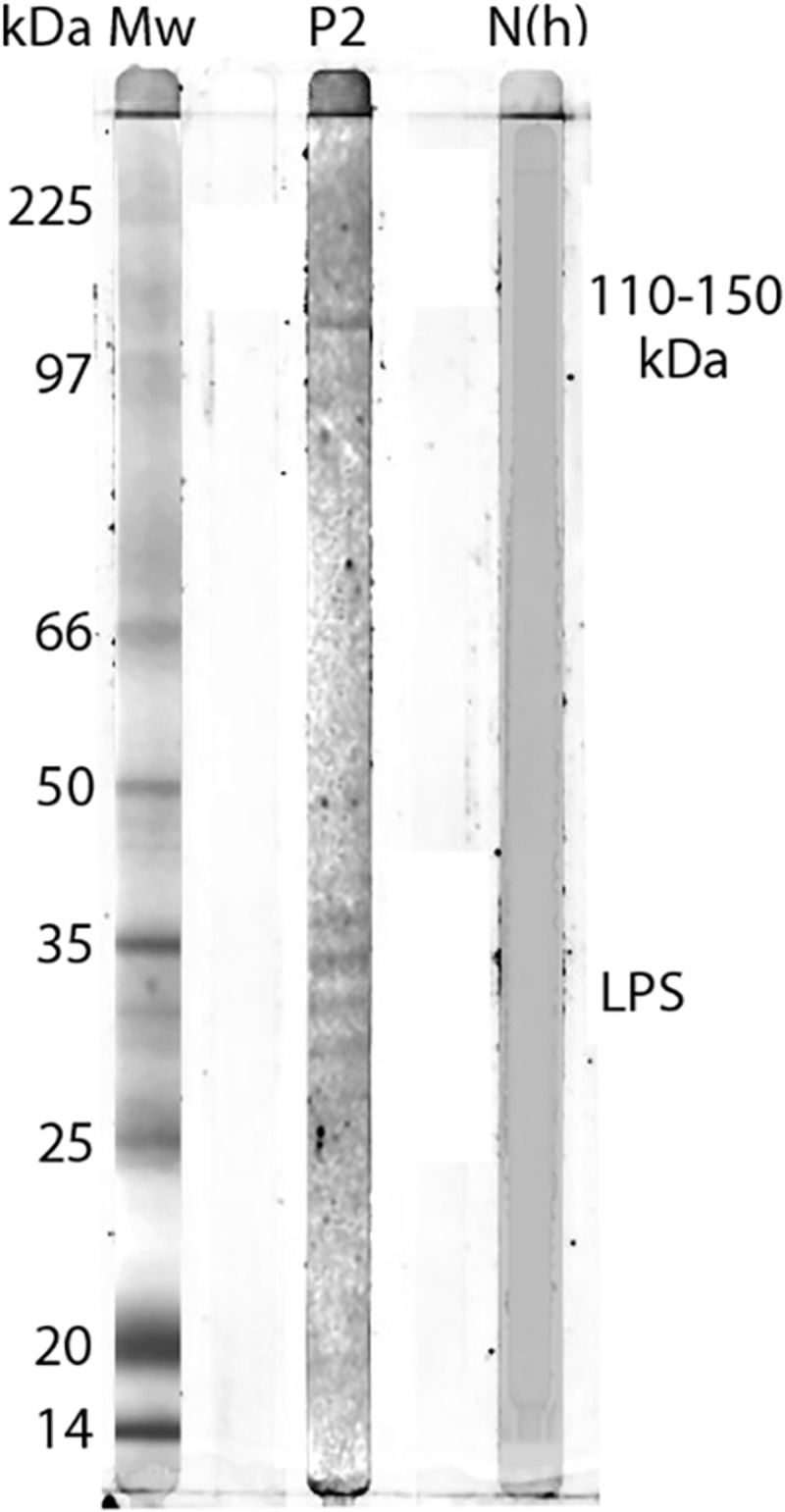
Western blot analysis of IgG antibodies against *R. africae* whole cell antigen. Lane P2 demonstrates the lipopolysaccharide ladders (LPS) and specific reactions against spotted fever rickettsia proteins in the 110–150 kDa region for convalescent serum for Patient 2.

Western blot for Patient 2 showed a specific response against lipopolysaccharide (LPS) and protein antigens in the 110–150 kDa region for IgG to whole cell antigen of *R. helvetica* and *R. africae* (Figure 2). Negative controls in the form of serum from a healthy blood donor and an IFA-negative patient showed no specific reactions.

Both patients were treated with doxycycline 100 mg per dose twice a day for 10 days within a week after symptom onset. Neither of the patients required hospitalization, and both showed slow clinical improvement with persistent mild fever and headache and feelings of illness for a few days.

## Discussion

ATBF is a relatively common disease in rural sub-Saharan Africa, especially in South Africa.

Diagnosis of ATBF outside endemic areas, Africa and the West Indies, is usually based on travel history and patients typically present with mild to moderate flu-like symptoms, associated with one or multiple inoculation eschars, enlarged lymph nodes close to the eschars, and a delayed antibody response.[17,18] According to the literature, a rash is observed in 30–88% of cases, typically a maculo- or papulovesicular discrete rash.[19,20] No fatal cases have been reported to date. Nevertheless, a more severe course has been described in elderly populations.[9] The attack rate of ATBF is high, above 30%, and it is common for more than one person in a group of travellers to fall ill. The reason for this is explained by the hunting strategy of the tick vector, which runs towards its host when an animal or a human is nearby.[9,20] Both reported patients had mild symptoms of fever, malaise and in one case, lymph node swelling. Bite marks were seen in both cases, but no rash. Both patients also presented a mild transient thrombocytopenia, which is a frequently reported laboratory finding. Other findings are elevated CRP and liver enzyme elevation, though the latter was not seen in the present cases.

Severe complications have also been reported, such as cranial or peripheral neuropathy, chronic fatigue, reactive arthritis, encephalitis, myocarditis and cellulitis after ATBF.[9] A confirmatory laboratory method is necessary to differentiate ATBF from other febrile illnesses. By tradition, microbiological diagnosis of rickettsial diseases relies primarily on serologic test results.[1,5] For serologic testing, the immunofluorescence test is the preferred method. However, serology can remain negative 10 days after symptom onset, and definitive diagnosis based on seroconversion cannot usually be made until a month later when a rise in the titre of IgG antibodies has occurred, an outcome also observed in the two patients studied here. It is also clear that the result is affected by whether heterologous or homologous antigen is used. Moreover, interpretation of the serological response is complicated by the fact that Patient 2 was tick-bitten in central Sweden a few days before symptom onset and therefore also might have developed an antibody response in the second serum to *R. helvetica* exposure, as this tick (*I. ricinus*) is endemic in that area. The use of PCR on skin biopsy samples from the eschar is a proposed method and was very successful in Patient 2. However, obtaining a skin biopsy is not always easy or possible, for example in children. As an alternative, the use of a non-invasive swab has proven to be effective in other reported cases.[21,22] Rickettsial DNA has been shown to be detectable using this technique as long as eschars persist, i.e. about three weeks.[21,22] It may be an advantage, if possible, to remove the eschar crust before circulating the swab at the base of the eschar, to yield a sufficient sample, and thereafter to store the sample at 4°C. Temperature, unlike storage time, has proven to have a significant effect on DNA yield.[22] At the laboratory, the swab may then be placed in a small amount of saline prior to extraction. In Patient 1, the swab still contained enough material for a positive PCR result, even though it had already been used for a general bacterial culture. In all cases, the isolation and characterization of the causative pathogen from the clinical sample constitute the definitive test, but this option is limited by the fact that only some laboratories have the experience and facilities required to grow rickettsiae.

In conclusion, findings from the two patients studied here confirm the usefulness of PCR in detecting the agent of ATBF in an early phase. They also show the usefulness of using swabs instead of biopsies to collect a representative sample, which facilitates and improves the management of febrile patients who have recently visited Africa.
